# MALE AND FEMALE PERSPECTIVES: EXPLORING THE USE OF PASSIVE BRAIN HEALTH TECHNOLOGY TO MEASURE GLYMPHATIC FUNCTION

**DOI:** 10.1093/geroni/igad104.3461

**Published:** 2023-12-21

**Authors:** Cyriah Carey, Erica Sappington, Mitchell Roberts, Paul Dagum, Jeffrey Iliff, Swati Rane Levendovszky, Michael Jaffee, Carla VandeWeerd

**Affiliations:** UF Health Precision Health Research Center, The Villages, Florida, United States; UF Health Precision Health Research Center, The Villages, Florida, United States; UF Health Precision Health Research Center, The Villages, Florida, United States; Applied Cognition Inc, Redwood City, California, United States; University of Washington, Seattle, Washington, United States; University of Washington Medical Center, Seattle, Washington, United States; University of Florida College of Medicine, Gainesville, Florida, United States; UF Health Precision Health Research Center, The Villages, Florida, United States

## Abstract

Older adults’ (OA) research participation is essential to gain insights into the causes, progression, and potential treatments of Alzheimer’s Disease (AD). Brain health technology, such as the Glymphatic Function (GF) Monitor, has the potential to measure the progression of AD. The GF Monitor is a multimodal in-ear dynamic impedance spectroscopy and electroencephalogram (EEG) system worn during sleep and records physiological signals: photoplethysmogram (PPG), bioimpedance (BioZ), and acceleration. This study aims to understand OA perceptions and perceived benefits of passive brain health technology such as the GF Monitor. Six focus groups (3 female, 3 male) were conducted with OA (N=48) between June-August 2022 in The Villages, Florida. Domains assessed included concerns for developing AD, health technology perceptions, and preferences for data/outcomes visualization. Focus groups were transcribed verbatim, and content analysis identified salient themes. Across genders, motivation to participate in brain health technology research was influenced by concerns of family history of AD. Females expressed that using the GF monitor “is a small sacrifice to pay” for data-driven interventions and “preventative measures.” Males value “receiving easily digestible results” that allow them to monitor their cognitive health. Overall participants desire passive brain health monitoring to “stay active” and “remain independent” while aging in place. Passive technology has the potential to engage OA in research. Participants value prevention, maintaining independence, and receiving comprehensive results. The GF monitor has the potential to harness these wishes to monitor and receive translational brain health findings.

